# Attention deficit hyperactivity disorder in adults : prevalence and association with addictive disorders

**DOI:** 10.1192/j.eurpsy.2024.785

**Published:** 2024-08-27

**Authors:** S. Bader, A. Karkni, W. Abbes

**Affiliations:** ^1^psychiatry, University Hospital of Gabes, Gabes, Tunisia

## Abstract

**Introduction:**

Attention deficit hyperactivity disorder (ADHD) is a neurobehavioral disorder witch assumed to be a disorder of childhoood but recently has been shown to persist into adulthood. As in children, core features of adult ADHD include inattention, impulsivity, and/or hyperactivity.Despite growing interest in adult ADHD, little is known about its prevalence or correlates.

**Objectives:**

We aimed to estimate the prevalence of ADHD in adult outpatient psychiatric care at the university hospital of Gabes (southern Tunisia) and to explore its association with addictive disorders.

**Methods:**

We conducted a cross-sectional, descriptive, and analytical observation study,in the outpatient psychiatry department of the Gabes university hospital, during the period ranging from 1/1/2023 to 30/06/2023. We used an anonymous pre-established information sheet exploring the socio-demographic, clinical, therapeutic data of the patients, lifestyle habits and substance use, the DSM-5 to classify diagnoses, CGI-S to rate the severity of overall mental illness, Fagerström test to assess the nicotine dependence, Adult ADHD Self-Report Scale (ASRS) in its validated Arabic version to screen ADHD and the Diagnostic Interview for ADHD in Adults (DIVA) to confirm the ADHD diagnosis. Data entry and analysis were performed using Statistical Package for Social Sciences (SPSS) version 21.0.

**Results:**

The response rate in our study was around 64.5%, 205 patients were included.The mean age of the patients was 48years ±14.9, the male/female ratio was 1.The estimated prevalence of adult ADHD according to the DIVA was 5.9% (male/female ratio=1/2). At the uni-variate study, significant associations were found between ADHD and the age category (p=0), the marital status (OR=0.14; CI [0.03- 0.55], p=0.003), theFagestrom score (p=0.01), cannabis consumption (OR=19; CI [1.8-201], p=0.018), psychotropic drugs consumption (OR=39; CI[3-196], p=0.02), self-harm behavior (OR=6.9, CI[1.9-26], p=0.01), excessive use of internet and screens (OR=38, CI[7-179],p=0). At the multivariate study, two determining factors were found: cannabis consumption(OR=8 [1- 58]; p=0.031), and the excessive use of internet and screens (OR=25 [4-144]; p=0).

**Conclusions:**

Regarding our findings and the important prevalence of the adult ADHD,more efforts are needed to increase the detection and treatment of this disorder, in order to set up an early intervention before major impairments and complications become irreversible.

**Disclosure of Interest:**

None Declared

